# Trocar Site HERnias After Bariatric Laparoscopic Surgery (HERBALS): a Prospective Cohort Study

**DOI:** 10.1007/s11695-020-04400-y

**Published:** 2020-01-16

**Authors:** Ioannis Karampinis, Eliette Lion, Svetlana Hetjens, Georgi Vassilev, Christian Galata, Christoph Reissfelder, Mirko Otto

**Affiliations:** 1 Department of Surgery, Universitätsmedizin Mann heim, Medical Faculty Mannheim, Heidelberg University, Mannheim, Germany; 2 Institute of Medical Statistic and Biomathematics, Universitätsmedizin Mannheim, Medical Faculty Mannheim, Heidelberg University, Mannheim, Germany

**Keywords:** Trocar site hernia, Port site hernia, Incisional hernia, Bariatric surgery, Surgical complications

## Abstract

**Background:**

The exact prevalence of trocar site hernias after bariatric procedures is not yet known. Recent metaanalysis data indicated concerning rates of up to 25%. We conducted a prospective cohort study to estimate the prevalence and analyze the role of fascia closure in the development of trocar hernias.

**Method:**

A total of 365 patients who were operated for obesity in our department between 2009 and 2018 were included. All patients were invited for a follow-up ultrasonography scan in order to detect abdominal wall defects. The role of intraoperative fascia closure in the development of trocar site hernias was evaluated, and a logistic regression analysis was performed to detect potential risk factors.

**Results:**

The overall prevalence of trocar hernias detected by ultrasonography was 34%. The prevalence of abdominal wall defects in patients who received a fascia closure was 37% compared with 34% in patients who did not receive a fascia closure (*p* = 0.37). The only factor that was associated with a higher risk for trocar site hernias was high excessive weight loss (*p* = 0.05).

**Conclusion:**

Trocar site hernias are an underestimated complication of minimally invasive, multiportal bariatric surgery, and the prevalence of asymptomatic hernias is probably higher than initially expected. In this study, fascia closure did not protect against trocar hernias. However, opposing evidence from similar trials suggests closing the fascia. This clinical problem should therefore be further assessed in a prospective randomized setting.

## Introduction

Apart from obesity, the indication for performing bariatric procedures has now expanded to non-obese patients with metabolic disorders (1). This will probably lead to bariatric operations as one of the most frequently carried out procedures in the next decade. The need for reducing perioperative morbidity and surgical complications is thus increasingly crucial.

One of the most neglected complications of bariatric surgery is trocar site hernias. There are several reasons for this neglect. First, most studies have traditionally focused on the excellent surgical results with the intention of improving the impact on weight loss and metabolism. Second, depending on the health care system, operated patients often lose contact with their surgeon and receive follow-up care by general practitioners who do not routinely evaluate for late surgical complications. However, the most important reason is probably that trocar site hernias are not visible or palpable in the bariatric patient (2). Therefore, an imaging modality has to be routinely applied in order to detect hernias in this population, adding a significant burden of labor and costs to normal follow-up care in bariatric patients.

The precise estimation of the prevalence of trocar site hernias after laparoscopic surgery in bariatric patients is yet not possible, since no prospective randomized studies with long-term follow-up data have addressed this issue. A recent metaanalysis pooled data from retrospective studies and several prospective cohort studies in order to assess the prevalence of trocar site hernias and estimate the severity of the problem. After limiting our focus on studies particularly dedicated to the field of bariatric surgery, we estimated a concerning prevalence rate at 15% (3). One well-conducted study included in our analysis even reported prevalence levels reaching 40% (4).

The purpose of this study was to detect the prevalence of trocar site hernias after bariatric procedures. Furthermore, we focused on the role of fascia closure and evaluated for further risk factors that could influence the development of trocar site hernias.

## Materials and Methods

The study was approved by the local ethics committee and registered in the German clinical trials register (DRKS00013122) and has been performed in accordance with the ethical standards as laid down in the 1964 Declaration of Helsinki and its later amendments. All patients provided informed consent for participating in the study and the use of their data.

We included adult patients who had been operated in our department between 2009 and 2018. The minimum follow-up for inclusion in our study was set at 9 months after surgery. This time frame was established in order to avoid confusing the abdominal defects with postoperative changes caused by the healing process of the abdominal wall. Exclusion criteria included the following: laparoscopic surgery in the upper abdomen before the bariatric procedure, any type of midline or transverse laparotomy in the upper abdomen before or after the bariatric procedure, and procedures implementing implantable devices penetrating the fascial layer. We first analyzed the effect of fascia closure in the entire cohort by dividing the patients into two groups based on whether or not the fascia had been sutured during the bariatric operation. In order to define the absolute prevalence of trocar site hernias and the role of fascia closure in the development of trocar site hernias, we then repeated the statistical analysis including patients who had only had a single bariatric operation.

Surgery was always performed under general anesthesia. The pre-, peri-, and postoperative surgical care was standardized for all patients and was based on an updated version of a clinical pathway, which we have previously published (5). The fascia closure was performed using absorbable, synthetic, polyfilament, interrupted, braided stiches (polyglactin, strength 0) under camera visualization (bird pick technique). Only 12-mm trocar sites were closed. The fascia in 5-mm trocar sites was never closed. During sleeve gastrectomy, the middle 12-mm trocar was bluntly widened in order to enable the extraction of the resected stomach; the fascia at this site was always closed. These patients were allocated to the fascia closure group if the fascia at the remaining 12-mm trocar sites was closed and otherwise to the no fascia closure group if the fascia at the remaining 12-mm trocar sites was not closed. The trocar hernias at the extraction site for the stomach are presented descriptively, since no formal statistical analysis was possible.

Patients were examined using ultrasonography scans of the abdomen in order to detect trocar site hernias. All scans were performed by the same two examiners, who were experienced in abdominal sonography. In order to perform the ultrasonography, all trocar scars were identified and marked. In a radius of 5 cm using the trocar scar as the center, we searched for the subcutaneous insertion canal. All abdominal layers were identified. The dehiscence/hernia was defined as an interruption of the continuity of the fascia affecting all underlying layers up to the abdominal cavity.

**Fig. 1:**
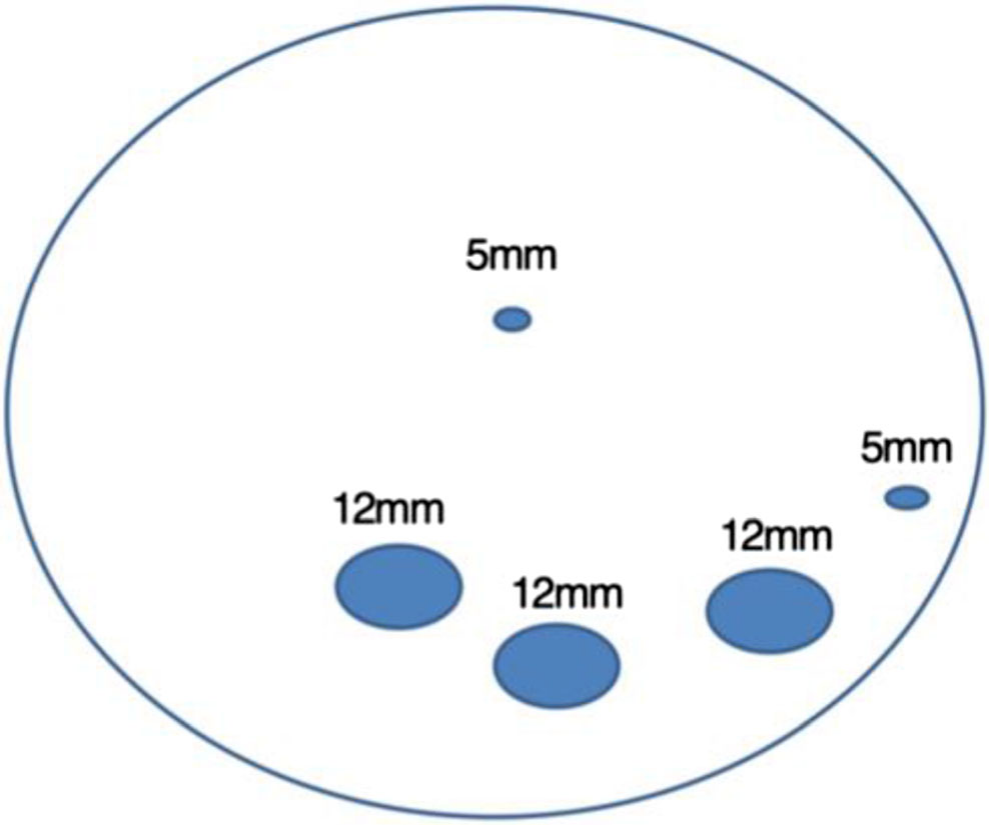
Standard trocar placement

Every trocar dehiscence was measured and documented (Fig. 1) for every patient. The trocars marked in this figure represent the standard trocar placement in our clinic. In cases of previous plastic reconstruction of the abdominal wall, we directly searched for the insertion canal through the fascia without taking the laparoscopy scars into account. The interobserver variability was tested before the beginning of this study and in random cases throughout the study, in order to assure maximum quality and precision of the sonography scan. During this process, the one examiner was blinded to the findings of the other. During every sonography scan, the examiner was blinded to the type of surgery and to the type of fascia closure.

### Statistics

All statistical calculations were performed using the SAS software, release 9.4 (SAS Institute Inc., Cary, NC, USA). Quantitative parameters are presented by median and range. Qualitative data are described by their absolute and relative frequencies. In order to compare groups for the analysis of qualitative parameters, a chi-square test or Fisher’s exact test was used, where appropriate. The mean values of two subgroups were compared using two-sample *t* tests (in the case of normally distributed data) or the Mann-Whitney *U* test. The logistic regression was performed as a multivariable model, in order to analyze two factors for a binary outcome simultaneously. The data were entered twice for 15 patients. To compare the results, the kappa index and the correlation coefficients (Pearson and Spearman) were calculated as a measure of agreement. Each test result with a value of *p* < 0.05 was considered statistically significant. The excessive weight loss was calculated according to the following formula: pre-op weight − current weight/pre-op weight-ideal weight (BMI 25).

## Results

Three hundred sixty-one patients were included and data from 359 patients were available for analysis. Two patients were excluded, one because of the short follow-up (4 months) and one because of the use of a robotic surgical approach. The overall follow-up rate was 56%. Patient characteristics are presented in detail in Table 1. The kappa coefficient and the correlation coefficients showed absolute conformity between the two examiners.

**Table 1:** Patients’ characteristics (stratified by fascia closure)

	Fascia closure (*n* = 73)	No fascia closure (*n* = 286)	*p*
Gender	54 F/19 M	225 F/61 M	0.38
Age (years)	44 ± 10 (range 21–64)	43 ± 12 (range 21–75)	0.48
BMI (at surgery)	51 ± 9 (range 35–80)	49 ± 7 (range 31–75)	0.06
BMI (at follow-up)	36 ± 8 (range 23–59)	35 ± 7 (range 21–55)	0.33
Smoking	29 (39.7%)	137 (47.9%)	0.24
DM II	29 (39.7%)	105 (36.7%)	0.68
Sleeve	23 (31.5%)	93 (32.5%)	0.93
RYGB	44 (60.3%)	181 (63.2%)	
Other	6 (8.2%)	12 (4.19%)	
TsH	27 (36.9%)	97 (33.9%)	0.58
ASA score			0.79
II	4 (5.5%)	20 (7%)	
III/IV	69 (94.5%)	266 (93%)	
LOS (min)	166 ± 64 (range 69–477)	110 ± 55 (range 44–556)	< 0.001
LOHS (days)	8 ± 2 (range 5–24)	6 ± 2 (range 3–23)	< 0.001
Follow-up (months)	81 ± 20 (range 38–119)	27 ± 18 (range 9–105)	< 0.001
Early complications			0.14
CD 0/I/II	68 (93.2%)	279 (96.9%)	
CD III/IV	5 (6.8%)	9 (3.1%)	
WHD	0	8 (2.8%)	0.17
EWL	0.61 ± 0.23 (range 0.06–1.13)	0.62 ± 0.24 (range − 0.60–1.62)	0.77

*F* female, *M* male, *BMI* body mass index (kg/m^2^), *DM II* type II diabetes mellitus, *RYGB* Roux-en-Y gastric bypass, *TsH* trocar site hernia, *ASA* American Society of Anesthesiology, *LOS* length of surgery, *LOHS* length of hospital stay, *CD* Clavien-Dindo classification, *WHD* wound healing disorders

**Fig. 2:**
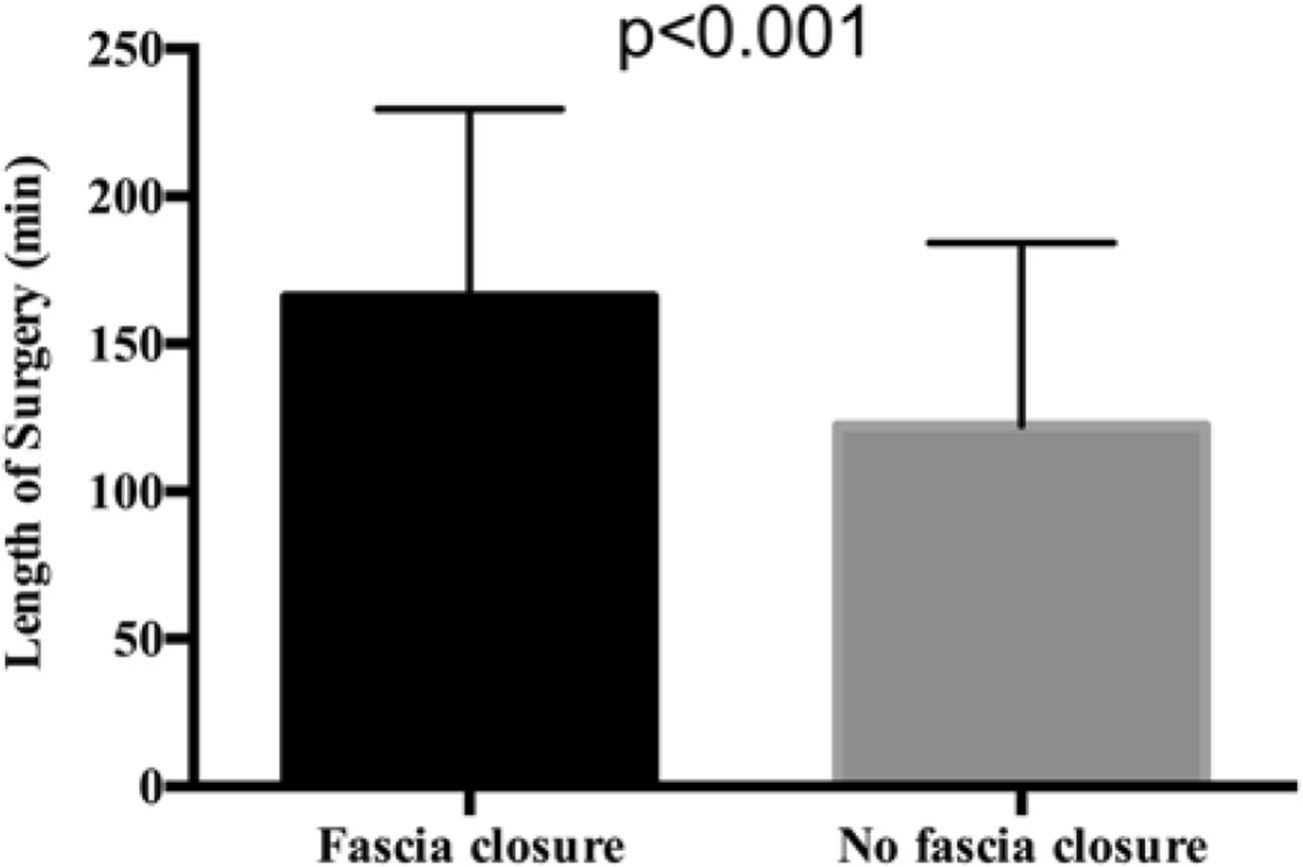
Length of surgery between the two groups

Three hundred thirty-one procedures were performed as a first bariatric approach, whereas in 28 cases, surgery was performed as a redo procedure. A trocar site hernia was detected in 124 patients (total prevalence 34.5%), and a total of 146 trocar site hernias were diagnosed in all 1795 trocar sites (8.13%). Patients’ characteristics did not differ significantly between the group who had received a fascia closure and the group where the fascia had not been closed; however, the length of surgery (Fig. 2), the length of hospital stay, and the length of follow-up were significantly different (Table 1). All the aforementioned parameters were longer in the group of patients where fascia closure was performed.

Due to the multiple trocar sites for each patient and the different operations with varying procedures at specific trocar sites (e.g., the extraction site after sleeve gastrectomy), we separated the trocar sites and repeated the analysis (Table 2).

**Table 2:** Distribution of trocar site hernias

	T1	T2	T3	T4	T5
Fascia closure	n.c.	12.3%	30%/22.4%*	5.5%	n.c.
No fascia closure	3.3%	7.7%	16%	7.3%	1.7%

T1: 5-mm trocar site left flank; T2: 12-mm trocar site left upper abdomen; T3: 12-mm supraumbilical trocar site and extraction site of the stomach in case of sleeve gastrectomy; T4: 12-mm trocar site in the right upper abdomen; T5: 5-mm trocar site epigastrium

*Represents hernias in the extraction site of the stomach

*n.c.*, no closure

### 5-mm Trocars

Eighteen cases of trocar site hernias were detected in the 5-mm trocar sites that were never closed in our department due to the previously established very low prevalence of hernias in trocar sites smaller than 10 mm. The overall hernia prevalence in the 5-mm trocar site was 2.5%. There were no patients with a symptomatic trocar hernia in this group, and there was one case of a wound healing disorder at a 5-mm trocar site in the left flank.

### 12-mm Trocars

**Fig. 3:**
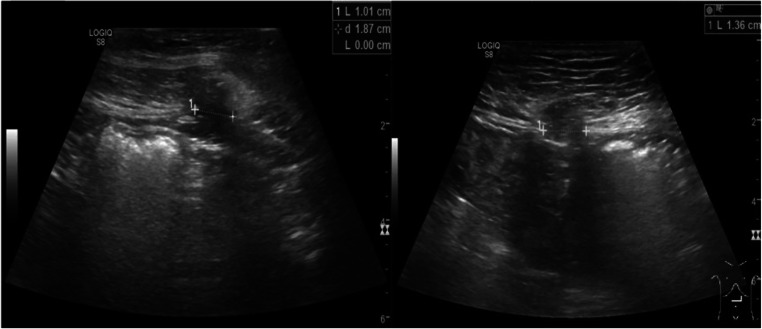
Sonography scan showing a trocar site hernia (left) and a further hernia with omental fat incarceration (right)

We then analyzed the 12-mm trocar sites, excluding those that had been widened to enable the extraction of the resected stomach. In 961 trocar sites, we detected 102 trocar hernias (10.6%, Fig. 3). There were six cases of wound healing disorders and four patients with symptomatic trocar site hernias with bowel incarceration who received an emergency operation.

### 12-mm Trocar Sites Used for Stomach Extraction

The trocar sites that were used for the extraction of the resected stomach after sleeve gastrectomy were analyzed separately. Twenty-six trocar site hernias were detected in 116 locations (22.4%). The fascial layer was always closed in this trocar site. There were two patients with wound healing disorders who did not require further surgical treatment, and no cases of symptomatic trocar site hernias. There was no significant difference in the number of trocar site hernias between the fascia closure group and the no fascia closure group.

The four cases of symptomatic trocar site hernias, in which an emergency operation was performed, were found to be directly caused by incarceration in one of the trocars sites used during the bariatric procedure. Additionally, nineteen cases of symptomatic trocar site hernias were observed during the follow-up period. These nineteen cases are not formally considered to be a long-term complication of the bariatric operation, as these patients had other laparoscopic operations before or after the bariatric procedure. We were thus unable to establish a direct causality between the bariatric operation and the development of the hernia.

### Patients Without Previous Operations

Due to the high probability of bias resulting from the multiple operations that most of our patients received before or between the first bariatric procedure and our follow-up ultrasonography, we isolated patients who were only operated once and had no other operations in the upper abdomen. Patients with non-bariatric surgery below the umbilical level were not excluded from analyses. After excluding patients with previous operations, 159 patients were available for inclusion. Of those, 36 patients had a fascia closure and in 123 cases, the fascia was not closed. BMI at surgery and BMI at follow-up were significantly higher in patients without fascia closure (*p* = 0.01 in both cases). However, excessive weight loss was not different between the two groups. Furthermore, patients with fascia closure experienced a significantly longer duration of the surgical procedure, length of hospital stay, and length of follow-up than patients without fascia closure (*p* < 0.001 for all 3 outcomes). Age, gender, smoking history, ASA score, and postoperative complications did not differ significantly between the two groups.

Fourteen trocar site hernias (38.8%) were found in the group of patients with fascia closure, and 35 trocar site hernias (28.4%) were detected in the group without fascia closure. No significant difference was detected (*p* = 0.23).

We performed a multivariate analysis in order to detect for potential risk factors associated with the development of trocar site hernias. Age, gender, BMI at surgery, BMI at follow-up, excessive weight loss, type of surgery, diabetes, smoking history, ASA score, abdominoplasty reconstruction, length of surgery, surgeon, and length of hospital stay were evaluated. The only factor that was associated with a higher risk for trocar site hernias was excessive weight loss (*p* = 0.05). Furthermore, we repeated the initial analysis comparing the demographics of patients who developed hernias with the patients who did not develop hernias (Table 3).

**Table 3:** Patients’ characteristics (stratified by the diagnosis of TsH)

	Hernias (*n* = 124)	No hernias (*n* = 233)	*p*
Gender	101 F/23 M	177 F/56 M	0.23
Age (years)	43 ± 10 (range 21–64)	43 ± 12 (range 21–75)	0.83
BMI (at surgery)	49 ± 8 (range 33–78)	50 ± 8 (range 31–80)	0.72
BMI (at follow-up)	35 ± 7 (range 23–57)	35 ± 7 (range 21–59)	0.30
Smoking	34 (27.42%)	76 (32.62%)	0.63
DM II	51 (41.1%)	78 (29.8%)	0.16
Sleeve	44 (31.5%)	70 (32.5%)	0.39
RYGB	72 (60.3%)	152 (63.2%)	
Other	8 (8.2%)	11 (4.19%)	
Fascia closure	27 (21.77%)	45 (19.31%)	0.58
ASA score			0.65
II	9 (7.3%)	14 (6%)	
III/IV	115 (92.7%)	219 (94%)	
LOS (min)	121 ± 63 (range 44–501)	121 ± 61 (range 48–556)	0.99
LOHS (days)	6 ± 2 (range 3–23)	6 ± 2 (range 4–24)	0.61
Follow-up (months)	36 ± 28 (range 9–119)	38 ± 28 (range 10–117)	0.41
Early complications			< 0.001
CD 0/I/II	120 (98.2%)	226 (65.3%)	
CD III/IV	4 (1.8%)	14 (34.7%)	
WHD	4 (3.22%)	3 (1.28%)	0.24
EWL	0.58 ± 0.25 (range − 0.60–1.13)	0.63 ± 0.24 (range − 0.09–1.62)	0.05

*F* female, M male, *BMI* body mass index (kg/m^2^), *DM II* type II diabetes mellitus, *RYGB* Roux-en-y gastric bypass, *TsH* trocar site hernia, *ASA* American Society of Anesthesiology, *LOS* length of surgery, *LOHS* length of hospital stay, *CD* Clavien-Dindo classification, *WHD* wound healing disorders

## Discussion

The precise estimation of the prevalence of the trocar site hernias after bariatric procedures is a challenge. The bariatric patient usually carries all known risk factors for developing trocar site hernias. Diabetes, limited physical condition, being overweight and a history of smoking are only some of the factors that have been associated with postoperative hernias (6). On the other hand, the particularly low prevalence of wound complications after bariatric surgery seems to play a protective role in reducing the risk for developing trocar site hernias.

Data from retrospective studies with adequate follow-up intervals indicate a notably high prevalence of trocar site hernias after bariatric surgery, ranging from 15 to 40% (2, 4, 7). However, apart from our results, the highest prevalence of trocar hernias was reported in a similar study by Scozzari et al., which provided the only documented systematic screening using imaging techniques for this particular population with regard to trocar site hernias up until now (4).

Suturing the fascia with interrupted, absorbable stiches did not result in fewer trocar site hernias in this study than leaving the fascia open. On the other hand, Rebibo et al. reported a significantly lower prevalence of trocar site hernias when the fascia was closed using a shielded port closure device (8). Furthermore, the multivariate analysis indicated excess weight loss (EWL) as the only risk factor for developing trocar site hernias. Higher EWL values were associated with a higher prevalence of postoperative hernias, which can be explained by the radical changes in the abdominal wall anatomy caused by extensive weight loss. One potential solution could be to change the suturing technique from interrupted to running sutures or to utilize a non-absorbable thread, which is the standard in closing laparotomies.

In this study, we detected a 34.5% rate of abdominal wall defects in a mean follow-up interval of 36.4 months. The primary challenge here is the clinical relevance, in terms of how many patients will eventually become symptomatic due to such defects. The prevalence in bariatric patients seems to be far higher than the reported prevalence in the non-bariatric population, which was estimated at 0.7% in a recent metaanalysis (9). Long-term surveillance data of patients with asymptomatic incisional hernias or trocar site hernias are not available, and the AWARE trial is currently evaluating relevant data (10).

Within our cohort, there were four patients who received an emergency operation for a small bowel herniation at the trocar site incisions, two of whom had a particularly complicated postoperative course. In these four cases, we could clearly identify the previous bariatric surgery as the cause of the hernia. Nevertheless, when analyzing the overall cohort, we discovered an additional nineteen patients who had developed symptomatic incisional hernias in the upper abdomen. In total, these cases result in an observed symptomatic hernia prevalence of 6.4%, but the real prevalence is probably much higher.

This finding results in a notable discrepancy between the sonographically detected abdominal wall defects and the ones that require surgery. Sonography scans can help in detecting abdominal wall defects, but routine implementation in the follow-up period for the bariatric patient requires significant resources and is still disputed. Therefore, targeted patient histories and clinical diagnostics evaluating for symptoms associated with postoperative hernias are crucial during follow-up visits, particularly in patients with substantial weight loss.

## Conclusion

The prevalence of symptomatic and asymptomatic trocar site hernias after bariatric surgery is probably much higher than previously assumed. Reducing the morbidity and mortality associated with trocar site hernias is essential. Both our earlier metaanalysis and this current study failed to demonstrate a difference in the prevalence of trocar site hernias between patients where the fascia was closed and in patients where the fascia was not closed. However, opposing evidence from similar trials suggests closing the fascia. This clinical problem should therefore be further assessed in a prospective randomized setting.

## Limitations

There are several limitations to this study. Despite the fact that patient inclusion and ultrasonography scans were performed prospectively, the bariatric operations and naturally the fascia closure took place in the past and were not performed in the context of a standardized prospective study. The prevalence of trocar hernias was estimated using sonography scans. This type of scan is recommended by the guidelines of the European Hernia Society (2015) for detecting abdominal wall defects. However, sonography cannot offer the same sensitivity rates with the CT scan (which on the other hand has a non-negligible amount of radiation). Nevertheless, both detection methods estimate the rate of abdominal wall defects and not of symptomatic hernias.

In this study, we included patients who received a bariatric operation over the course of nearly a decade. It is therefore important to note the evolution of bariatric surgery in our department and the changes in the learning curves and treatment algorithms, which can also explain the significant differences in operating time, length of hospital stay, etc., might have influenced the results of this study.

## References

[1] Mingrone G, Panunzi S, De Gaetano A, Guidone C, Iaconelli A, Nanni G, et al. Bariatric-metabolic surgery versus conventional medical treatment in obese patients with type 2 diabetes: 5 year follow-up of an open-label, single-centre, randomised controlled trial. Lancet (London, England). 2015 Sep 5;386(9997):964–73. Epub 2015/09/16. eng. 10.1016/S0140-6736(15)00075-6 26369473

[2] Ahlqvist S, Bjork D, Weisby L, Israelsson LA, Cengiz Y. Trocar site hernia after gastric bypass. Surgical technology international 2017 Jul 25;30:170–174. Epub 2017/07/12. eng. 28696492

[3] Karampinis I, Lion E, Grilli M, et al. Trocar site hernias in bariatric surgery-an underestimated issue: a qualitative systematic review and meta-analysis. Obes Surg. 2019 Jan;18 Epub 2019/01/20. eng 10.1007/s11695-018-03687-2 30659465

[4] Scozzari G, Zanini M, Cravero F, Passera R, Rebecchi F, Morino M. High prevalence of trocar site hernia after laparoscopic or robotic Roux-en-Y gastric bypass. Surg Endosc 2014 Oct;28(10):2890–2898. Epub 2014/05/03. eng. 10.1007/s00464-014-3543-5 24789133

[5] Ronellenfitsch Ulrich, Schwarzbach Matthias, Kring Anne, Kienle Peter, Post Stefan, Hasenberg Till (2012). The Effect of Clinical Pathways for Bariatric Surgery on Perioperative Quality of Care. Obesity Surgery.

[6] Walming S, Angenete E, Block M, Bock D, Gessler B, Haglind E. Retrospective review of risk factors for surgical wound dehiscence and incisional hernia. BMC Surg 2017 Feb 22;17(1):19. Pubmed Central PMCID: PMC5320761. Epub 2017/02/23. eng. 10.1186/s12893-017-0207-0 PMC5320761 28222776

[7] Rebibo L, Dhahri A, Chivot C, Cyril C, Yzet T, Regimbeau JM. Trocar site hernia after laparoscopic sleeve gastrectomy using a specific open laparoscopy technique. Surgery for obesity and related diseases : official journal of the American Society for Bariatric Surgery 2015 Jul-Aug;11(4):791–796. Epub 2015/04/13. eng. 10.1016/j.soard.2014.11.028 25863538

[8] Rebibo L, Demouron M, Dembinski J, et al. Impact of routine 12 mm epigastric trocar site closure on incisional hernia after sleeve gastrectomy: a prospective before/after study. Obes Surg. 2019 Jun;5 Epub 2019/06/07. eng 10.1007/s11695-019-03971-9 31168720

[9] Antoniou S. A., Morales-Conde S., Antoniou G. A., Granderath F. A., Berrevoet F., Muysoms F. E. (2015). Single-incision laparoscopic surgery through the umbilicus is associated with a higher incidence of trocar-site hernia than conventional laparoscopy: a meta-analysis of randomized controlled trials. Hernia.

[10] Lauscher JC, Leonhardt M, Martus P, Zur Hausen G, Aschenbrenner K, Zurbuchen U, et al. [Watchful waiting vs surgical repair of oligosymptomatic incisional hernias: current status of the AWARE study]. Chirurg. 2016 Jan;87(1):47–55. Epub 2015/05/15. Beobachtung vs. Operation oligosymptomatischer Narbenhernien : Aktueller Stand der AWARE-Studie. ger. 10.1007/s00104-015-0011-2 25971607

